# Ethnic inequalities in child stunting and feeding practices: results from surveys in thirteen countries from Latin America

**DOI:** 10.1186/s12939-020-01165-9

**Published:** 2020-04-09

**Authors:** Giovanna Gatica-Domínguez, Marilia Arndt Mesenburg, Aluisio J. D. Barros, Cesar G. Victora

**Affiliations:** 1 International Center for Equity in Health, Marechal Deodoro 1160, 3rd floor, Pelotas, RS Brazil; 2 Postgraduate Program in Epidemiology, Federal University of Pelotas, Pelotas, RS Brazil

**Keywords:** Stunting, Ethnic groups, Health equity, Health status disparities

## Abstract

**Background:**

Although the prevalence of child stunting is falling in Latin America, socioeconomic inequalities persist. However, there is limited evidence on ethnic disparities. We aimed to describe ethnic inequalities of stunting and feeding practices in thirteen Latin American countries using recent nationally representative surveys.

**Methods:**

We analyzed national surveys carried out since 2006. Based on self-reported ethnicity, skin color or language, children were classified into three categories: indigenous/ afrodescendant/reference group (European or mixed ancestry). Stunting was defined as height (length)-for-age < − 2 standard deviations relative to WHO standards. Family wealth was assessed through household asset indices. We compared mean length/height-for-age and prevalence of stunting among the three ethnic groups.

**Results:**

Thirteen surveys had information on indigenous and seven on afrodescendants. In all countries, the average length/height-for-age was significantly lower for indigenous, and in eleven countries there were significant differences in the prevalence of stunting: the pooled crude stunting prevalence ratio between indigenous and the reference group was 1.97 (95% CI 1.89; 2.05); after adjustment for wealth and place of residence, prevalence remained higher among indigenous (PR = 1.34, 95% CI 1.28; 1.39) in eight countries. Indigenous aged 6–23 months were more likely to be breastfed, but with poor complementary feeding, particularly in terms of dietary diversity. Afrodescendants showed few differences in height, and in two countries tended to be taller compared to the reference group.

**Conclusions:**

In all Latin American countries studied, indigenous tended to be shorter and afrodescendants presented few differences with relation to the reference group. In order to reach the SDG’s challenge of leaving no one behind, indigenous need to be prioritized.

## Introduction

Linear growth is widely regarded as an excellent marker of child health and well-being [1]. In low and middle-income countries (LMICs), linear growth faltering is particularly pronounced during the window of the first 1000 days, from conception to the second birthday [2]. The main immediate causes of growth faltering, and thus of child stunting include poor diets, inadequate care, and infectious diseases. Poverty is the major distal determinant of stunting, acting through the above-mentioned immediate causes [1]. The socioeconomic determination of child stunting is made evident by the strong wealth-related inequalities that are present in most LMICs [3–6].

The short-term consequences of stunting carry both direct social costs (e.g., higher mortality and morbidity) and indirect costs including reduced cognitive development and higher rates of school dropout and grade retention. Stunting may lead to irreversible, long-term reductions in human capital including adult height, intelligence, school achievement, economic productivity, among others [5, 7, 8]. These short- and long-term consequences contribute to the perpetuation of the vicious cycle of poverty and inequalities [7]. The importance of child stunting has been recognized by the World Health Organization (WHO) in 2012, when its member states adopted resolution WHA 65.6, which endorses a global target of reducing by 40% the number of stunted under-five children by 2025. Stunting was also included in the Sustainable Development Goals as indicator number 2.2.1 (https://sustainabledevelopment.un.org/). In the context of Latin America and the Caribbean, stunting prevalence was reduced by 7.3 percentage points, from 16.9% in 2000 to 9.6% in 2017. Despite this reduction, stunting remains a major public health problem in the Latin American region, with 5.1 million children affected in 2017 [9]. Given the social determination of stunting, these children are particularly concentrated among poor and rural families, where the prevalence is substantially above what is observed at the national level [5].

In contrast to the ample evidence on socioeconomic inequalities in stunting, data on ethnic inequalities are still scarce in Latin America and the Caribbean, where the current population stems from three major ethnic groups, namely indigenous, afrodescendants and Europeans, with considerable degrees of miscegenation. Historically, indigenous families have been the most disadvantaged ethnic group in many countries of the Latin American region [10], and their children present a high prevalence of stunting [11–18]. Regarding afrodescendant children, we were unable to identify any multi-country analyses of stunting prevalence, in spite of the persistence of historical socioeconomic exclusion of the descendants of Africa slaves [19].

SDG 17.18 stresses the need for national statistics stratified according to “income, gender, age, race, ethnicity, migratory status, disability, geographic location and other characteristics relevant in national contexts.” To address this need, and to fill an important gap in the literature, we describe ethnic inequalities in stunting and feeding practices in thirteen Latin American countries using recent nationally representative surveys. We give particular emphasis to the intersectionality between ethnicity and wealth, and ethnicity and place of residence in the social determination of child stunting in the Latin American region.

## Methods

Our study design was a multi-country, cross-sectional survey analysis. We analyzed thirteen nationally representative surveys from Latin American countries: four Demographic Health Surveys or DHS (https://dhsprogram.com/ - Bolivia 2008, Colombia 2010, Guatemala 2014 and Honduras 2011), five Multiple Indicator Cluster Survey or MICS (http://mics.unicef.org/ - Belize 2015, Guyana 2014, Mexico 2015, Paraguay 2016 and Suriname 2010), one Reproductive health Survey or RHS (http://ghdx.healthdata.org/series/reproductive-health-survey-rhs - Nicaragua 2006) and three modified version of DHS in Brazil (Pesquisa Nacional de Demografia e Saúde da Criança e da Mulher 2006), Ecuador (Encuesta Nacional de Salud y Nutrición 2012) and Peru (Encuesta Demográfica y de Salud Familiar 2016). These global survey agencies use similar methodology and measurement protocols due to close collaboration and work through interagency processes, which facilitates the comparison of results [20]. All surveys included in the analyses rely on multistage sampling procedures, selecting regions within countries, administrative units within each region (e.g. municipalities), census tracts within each administrative unit, and households within each tract. In the selected households, all women aged 15–49 years are invited for an interview. and children aged up to 59 months are measured and weighted using obtain standardized anthropometric equipment. Further information on their methodologies are available in the published national reports [21–33].

Information on ethnicity or a proxy (i.e., skin color or language) from women in DHS and RHS, or for the heads of households in MICS was obtained. We used three categories in the analyses, namely indigenous, afrodescendants and the reference group, as presented in Additional file 1: Supplementary Table 1. We classify women as part of the reference group because they did not declare themselves as either indigenous or afrodescendants. In many countries, the reference group includes privileged subgroups in terms of socioeconomic and health indicators, but in other countries this group mostly includes individuals with mixed European and indigenous, or possibly some African, ancestry. It is important to note that Suriname and Guyana had special classifications for the reference groups, because it was not possible to identify a subgroup with an European background, and Asians (East Indians and Javanese) were also included in this category. Further information on our classification was published elsewhere [34].

Supine length for children aged up to 23 months and standing height for children aged 24–59 months were obtained by trained personnel using infantometers and stadiometers, respectively. The measuring boards used to collect anthropometric measures included ShorrBoards®, Seca® 217, or locally manufactured boards. We re-analyzed each survey to calculate length/height-for-age Z scores using a STATA macro (https://www.who.int/childgrowth/software/en/), derived from recommended methodology and data quality assessment by WHO and UNICEF. Z scores were considered as valid when these ranged from − 6 to 6 [35]. Under-five children were classified as stunted if they presented a length- or height-for-age Z score < − 2 standard deviations from the median of the WHO 2006 child growth standards for their sex. The continuous length- or height-for-age Z score values were also used to draw frequency distribution graphs for ethnic groups.

Linear growth faltering, that results in stunting, is most pronounced for the age range between 6 and 23 months; this is the age range for which international organizations prioritize measurement of dietary quality [16]. We calculated prevalences of three complementary feeding indicators in the 24 h preceding before the survey for children in this age range, according to the 2008 WHO definitions: 1) minimum dietary diversity – proportion of children who received foods from four or more out of seven food groups; 2) minimum meal frequency – proportion of children breastfed who received two or more (age 6–8 months) or three or more (age 9–23 months) feeds a day, including solid, semi-solid, or soft foods, plus the proportion of non-breastfed children who received four or more milk feeds; and finally, 3) minimum acceptable diet – proportion of breastfed children who had at least the minimum dietary diversity and the minimum meal frequency, plus the proportion of non-breastfed children who received at least two milk feedings, plus at least the minimum dietary diversity (not including milk feeds) and at least the minimum meal frequency. We also report on the prevalence of breastfeeding in the 24 h preceding the survey for the same age range.

DHS and MICS datasets provided household asset scores generated through principal component analysis (PCA). The analysis uses information on household goods, building materials and utilities like water and electricity, which are adjusted for urban or rural residence according to a methodology developed by DHS [36]. The first component of the PCA was used as a continuous variable in the regression analysis, and was also used to classify households into wealth tertiles. This classification was used in place of the more frequently used wealth quintiles given the limitations in sample size when stratifying by ethnic subgroups. Urban and rural residence was based on the classifications adopted by national governments at the time of the survey. Data used in our analyses are publicly available and ethical approvals were handled by the institutions carrying out the surveys.

Stunting prevalence was described by country and ethnic group. Using Poisson regression [37], we obtained stunting prevalence ratios comparing indigenous or afrodescendants with reference children. Crude and adjusted (by wealth index and place of residence) metanalyses were conducted to estimate the overall prevalence ratio, and the heterogeneity test (I^2^) to assess variability in the estimates across countries. The I^2^ ranges between 0 and 100%, and to classify the magnitude of heterogeneity we used the following classification: homogeneous (≤25%), moderately heterogeneous (26–74%) and highly heterogeneous (≥ 75%) [38]. To assess whether the type of survey (DHS, MICS or RHS) had an impact on the findings, we carried out meta-regression including this variable.

Univariate kernel density graphs [39] of length/height-for-age Z scores were used to produce smooth distribution curves for the comparison of indigenous versus reference children, and afrodescendants versus reference children. We assessed differences between distributions using the Student’s t-test. We also carried out intersectionality analyses by stratifying for both ethnicity and wealth tertiles, and for ethnicity and urban-rural residence. Because some of the combinations in the intersectionality analyses resulted in small sample sizes, we arbitrarily excluded estimates for which the sample size of stunting prevalence was lower than 25 observations in any given cell, in accordance with the standard practice of DHS reporting [40]. In order to assess socioeconomic inequalities within ethnic groups, we calculated the slope inequality index (SII) through a logistic regression model which uses the natural logarithm of the odds of stunting prevalence as the outcome and the wealth tertiles as the independent variable. The SII represents the absolute difference in the fitted value of stunting prevalence between the highest and the lowest values of the wealth index scale [41]. Interaction tests between ethnicity and place of residence were also performed. All analyses were carried out in Stata 15 (StataCorp, College Station, TX, USA), by taking into account the survey design, including sampling weights, clustering and stratification.

Ethical clearance was obtained by the institutions that conducted the surveys in each country.

## Results

Thirteen countries had surveys carried out since 2006 (median year 2012) with information on ethnicity and on stunting prevalence. All thirteen surveys had information on indigenous children and seven also had information on afrodescendants. The largest proportions of indigenous population were found in Bolivia and Guatemala (65.3 and 46.7%, respectively), whereas Brazil, Paraguay and Suriname (1.6, 2.8 and 4.7%, respectively) presented the lowest percentages. Brazil and Suriname had the largest proportions of afrodescendants (62.8% and 55.6, respectively), while the lowest frequencies were in Honduras (2.4%) and Ecuador (4.6%).

The distribution of indigenous, afrodescendants and reference children according to wealth tertiles and urban-rural residence are shown in Additional file 2: Supplementary Figures 1 and 2, respectively. In all countries, indigenous children tended to belong to poorer families than afrodescendant and reference children. In three countries of the seven countries with information, afrodescendants were poorer than the reference group (Brazil, Colombia, Ecuador and Suriname) whereas in the remaining four countries the opposite was observed (Belize, Guyana and Honduras). Regarding place of residence, indigenous families were more likely to live in rural areas than the other two ethnic groups in all countries. In contrast, afrodescendants were more likely to have urban residence than the reference group, except in Guyana.

Table 1 shows that national sample sizes varied from 2418 children in Belize to 21,253 in Peru, whereas stunting prevalence ranged from 5.6% in Paraguay to 46.7% in Guatemala. When data were disaggregated by ethnic group, the smallest sample size was observed for indigenous children in Brazil and Suriname (111 and 118 children, respectively). All other categories had more than 250 children for analyses. Indigenous children were more stunted than reference children. Guatemala had the highest prevalence of stunted indigenous children (61.4%) and was the country with the greatest inequality between indigenous and reference children. Brazil and Suriname presented the lowest stunting prevalence among indigenous children (12.4 and 12.1%, respectively), as well as the lowest inequalities in stunting between indigenous and reference children. Afrodescendant children were slightly less stunted than reference children, with the exception of Suriname and Brazil. However, these two countries had the lowest stunting inequality between afrodescendant and reference children.

**Table 1:** Sample sizes and stunting prevalence among children under the age of five years, by ethnicity and country

Country/year	N	Stunting (%)	95% CI	Ethnic group	N	Stunting (%)	95% CI
Belize 2015	2418	14.9	(13.0;17.1)	Indigenous	379	34.7	(27.9; 42.3)
				Afrodescendant	677	8.6	(6.3; 11.6)
				Reference	1133	14.0	(12.0; 16.3)
Bolivia 2008	8325	27.1	(25.5; 28.7)	Indigenous	4489	33.1	(31.1; 35.2)
				Afrodescendant	–	–	–
				Reference	3203	16.1	(14.2; 18.2)
Brazil 2006	4389	7.3	(6.0; 8.8)	Indigenous	111	12.4	(4.9; 28.3)
				Afrodescendant	2650	7.7	(5.9; 9.9)
				Reference	1463	6.5	(4.9; 8.6)
Colombia 2010	17,784	12.6	(11.9; 13.4)	Indigenous	2374	27.4	(23.3; 31.8)
				Afrodescendant	1918	10.3	(8.6; 12.3)
				Reference	11,421	11.8	(11.1; 12.7)
Ecuador 2012	8206	24.6	(23.0; 26.4)	Indigenous	1170	41.8	(37.0; 46.7)
				Afrodescendant	319	16.9	(11.4; 24.4)
				Reference	6464	23.6	(21.7; 25.6)
Guatemala 2014	12,259	46.7	(45.0; 48.4)	Indigenous	5109	61.4	(59.0; 63.7)
				Afrodescendant	–	–	–
				Reference	6588	34.1	(32.1; 36.1)
Guyana 2014	2997	11.3	(9.6; 13.2)	Indigenous	603	24.4	(19.7; 30.0)
				Afrodescendant	796	6.6	(4.4; 9.8)
				Reference	1585	10.7	(8.7; 13.1)
Honduras 2011	10,926	22.7	(21.5; 23.9)	Indigenous	1618	32.4	(29.1; 35.9)
				Afrodescendant	257	14.2	(9.9; 20.0)
				Reference	7764	22.2	(20.8; 23.5)
Mexico 2015	7855	12.4	(10.6; 14.3)	Indigenous	824	25.2	(20.0; 31.3)
				Afrodescendant	–	–	–
				Reference	7023	11.2	(9.4; 13.2)
Nicaragua 2006	6584	20.7	(19.0; 22.5)	Indigenous	355	30.4	(25.1; 36.4)
				Afrodescendant	–	–	–
				Reference	5790	20.0	(18.3; 21.9)
Paraguay 2016	4419	5.6	(4.6; 6.8)	Indigenous	268	31.3	(19.1; 46.8)
				Afrodescendant	–	–	–
				Reference	4151	4.9	(4.0; 5.8)
Peru 2016	21,253	13.1	(12.3; 13.9)	Indigenous	1719	33.8	(30.6; 37.1)
				Afrodescendant	–	–	–
				Reference	17,448	11.5	(10.7; 12.3)
Suriname 2010	2710	8.8	(7.6; 10.2)	Indigenous	186	12.1	(7.8; 18.4)
				Afrodescendant	1711	9.3	(7.6; 11.2)
				Reference	791	8.0	(6.1; 10.4)

**Fig. 1:**
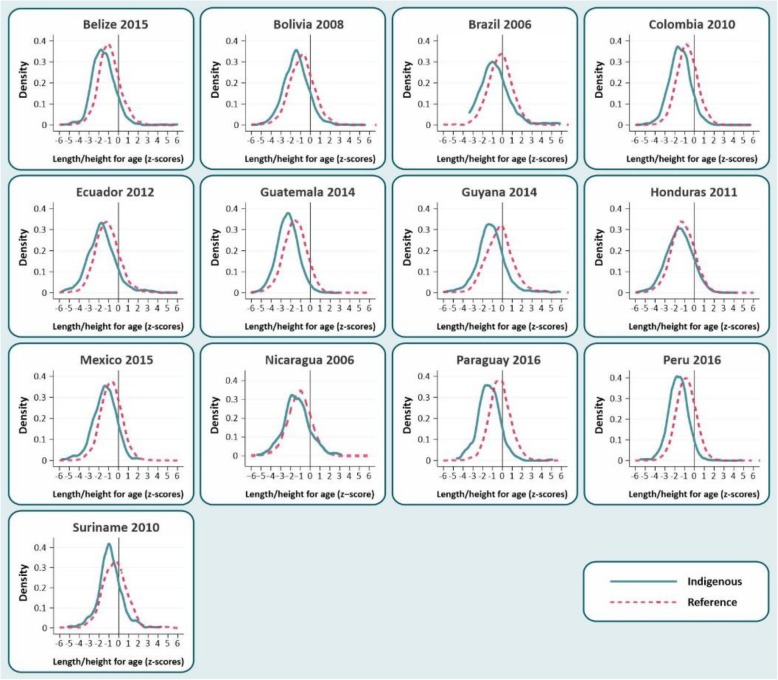
Univariate kernel density graphs of length/height-for-age Z scores of indigenous versus reference children from thirteen Latin American countries

Figure 1 shows the univariate kernel density graphs of length/height-for-age Z scores for indigenous and reference children. In all countries, the length/height distribution of indigenous children is shifted to the left compared to the WHO standard (which has a mean value of zero) as well as to the reference children. The length/height-for-age distributions of reference children from Brazil, Guyana, Paraguay and Suriname were superimposed with the WHO standard, but in all other countries there is evidence of a shift to the left even in the reference groups. The length/height-for-age distributions were statistically different between indigenous and reference children in all countries (*p* < 0.05).

**Fig. 2:**
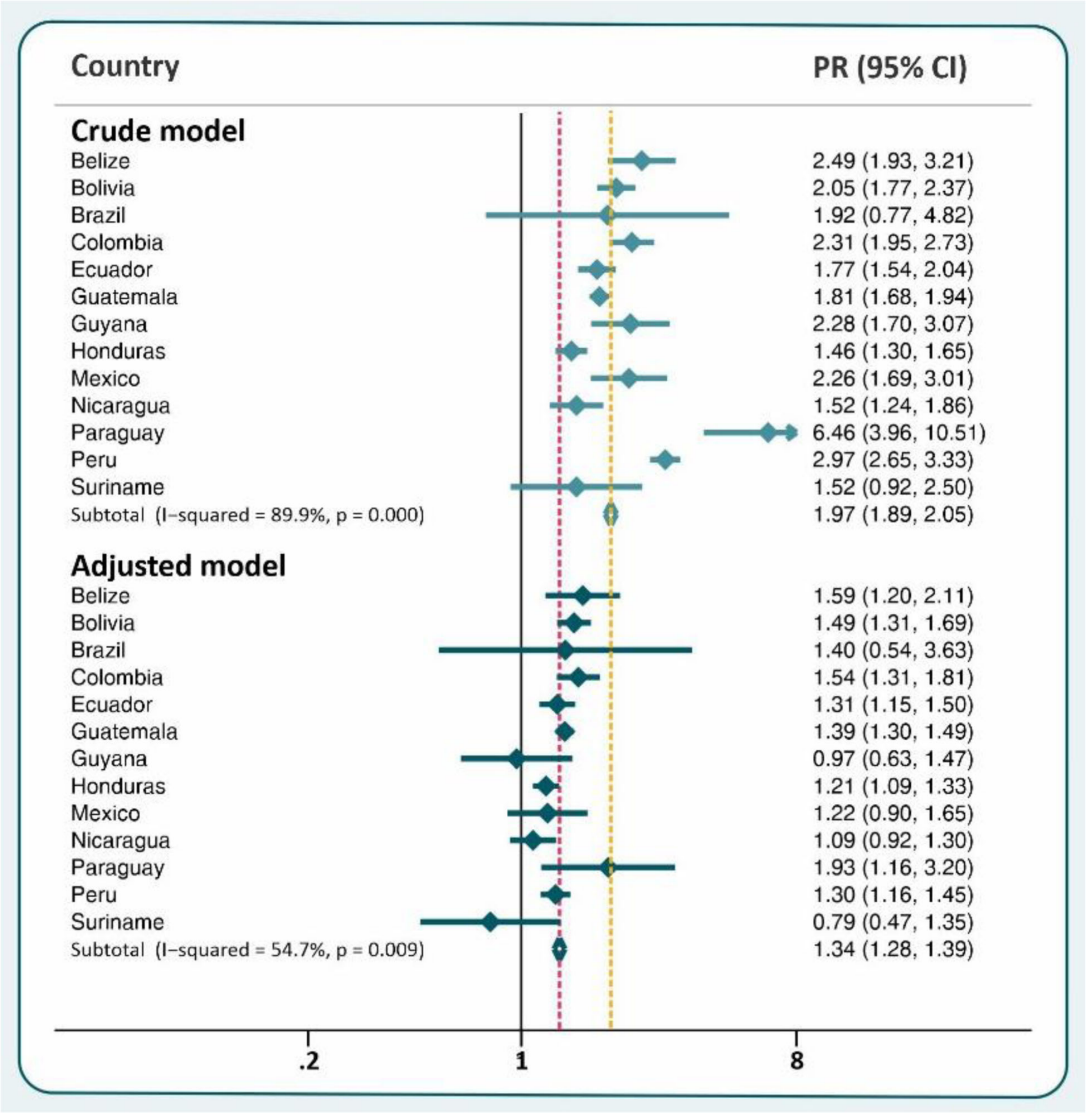
Stunting prevalence ratio for indigenous children compared to the reference group, showing crude and adjusted (by wealth index and urban-rural residence) results

Stunting prevalence ratios comparing indigenous and reference-group children derived through Poisson regression are shown in Fig. 2. In the unadjusted analyses, all thirteen countries showed higher stunting prevalence among indigenous children than in the reference groups, but in Brazil and Suriname the 95% confidence interval included the null value of 1. These were the two countries with the smallest proportions of indigenous children, as noted above. The pooled crude stunting prevalence ratio between indigenous and reference children was 1.97 (95% CI 1.89; 2.05) with evidence of heterogeneity across countries (I^2^ = 89.9%). After adjustment for wealth and residence all prevalence ratios were attenuated, and for Guyana, Mexico and Nicaragua the 95% confidence intervals included the null value of 1. The overall adjusted stunting prevalence ratio between indigenous and reference children was 1.34 (95% CI 1.28; 1.39) with moderate heterogeneity across countries (I^2^ = 54.7%), and the type of survey only explains 6% of the heterogeneity.

**Fig. 3:**
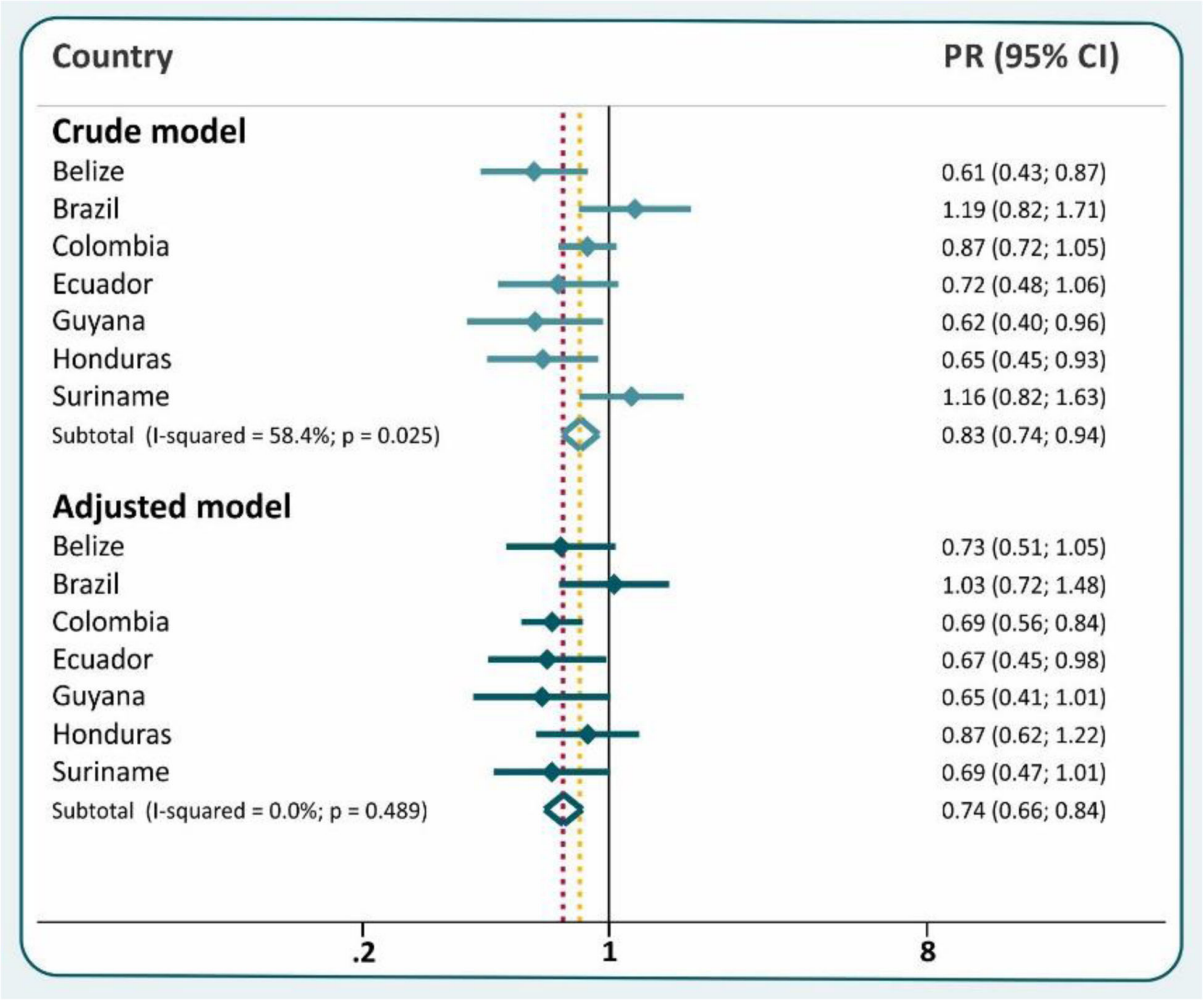
Stunting prevalence ratio for afrodescendant children compared to the reference group, showing crude and adjusted (by wealth index and urban-rural residence) results

Differences among afrodescendant and reference children were not marked (Fig. 3), and in general stunting prevalences tended to be lower among the former. In three countries (Belize, Guyana and Honduras), crude prevalence ratios were below 1.0 and the confidence interval did not include the null value of 1. The pooled crude stunting prevalence ratio between afrodescendant and reference children was 0.83 (95% CI 0.74; 0.94), and the heterogeneity across countries was moderate (I^2^ = 58.4%). After adjustment, afrodescendants showed lower prevalence than the reference children in Colombia and Ecuador. The overall adjusted stunting prevalence ratio between afrodescendant and reference children was 0.74 (95% CI 0.66; 0.84) with no evidence of heterogeneity across countries (I^2^ = 0%). In the meta-regression, the type of survey did not affect the comparison between afrodescendants and the reference group (adjusted R^2^ = 0%).

**Fig. 4:**
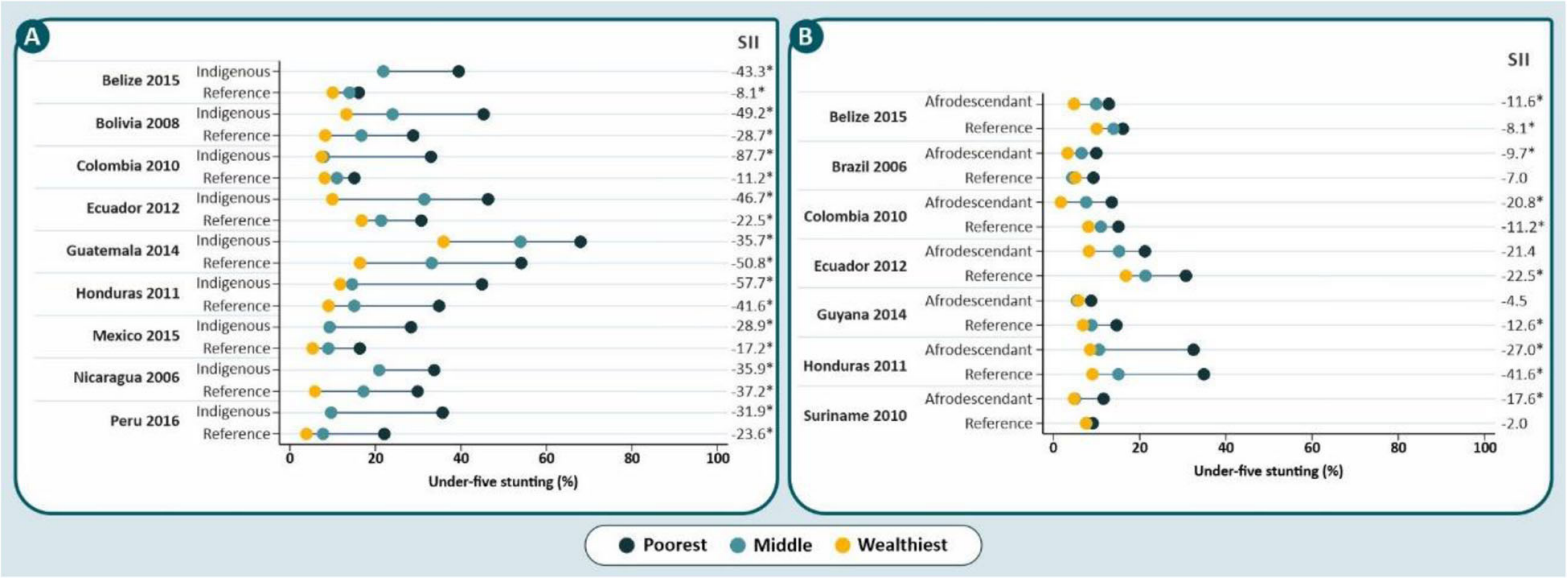
Stunting prevalence in indigenous (**a**) and afrodescendant (**b**) children from Latin American countries by wealth tertiles. Indigenous children from the richest tertile of Belize, Mexico, Nicaragua and Peru were excluded for including fewer than 25 observations (*) SII values with *P* < 0.05.

**Fig. 5:**
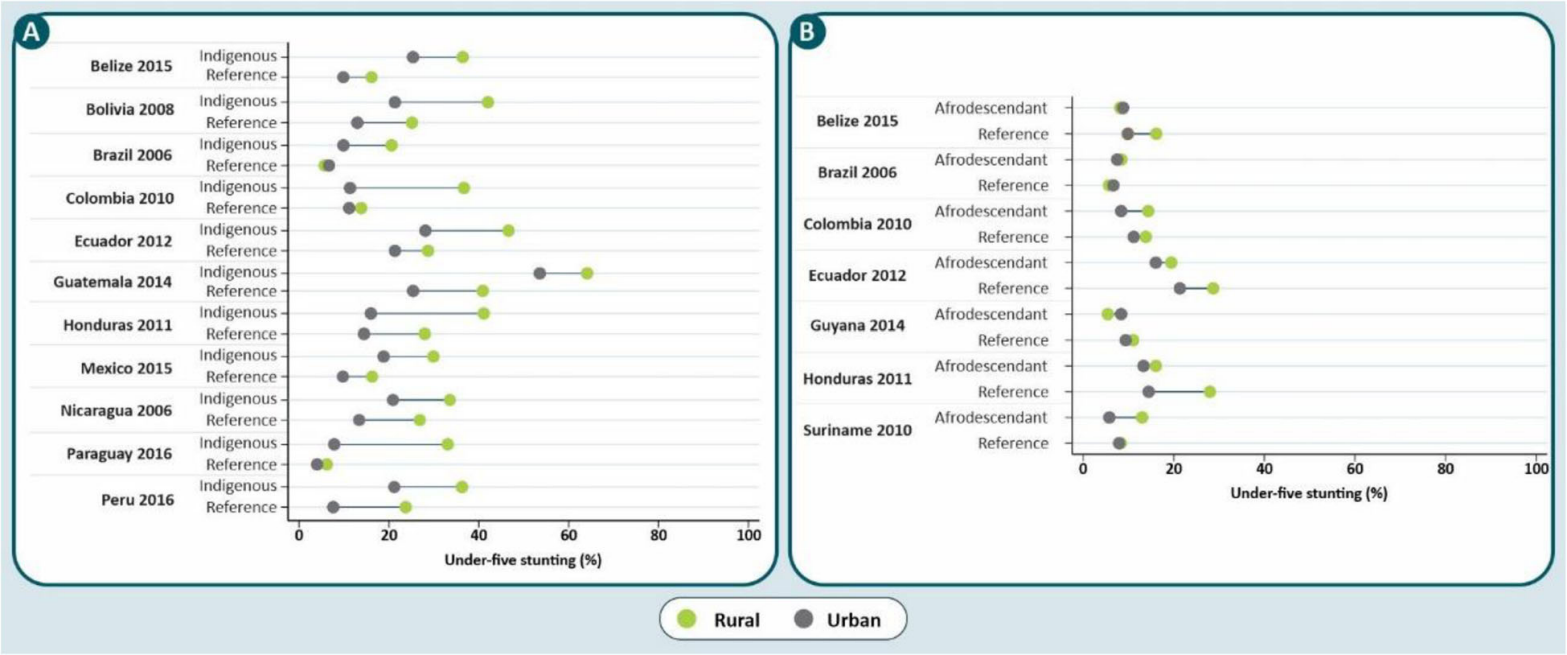
Stunting prevalence in indigenous (**a**) and afrodescendant (**b**) children from Latin American countries by place of residence

Figures 4 and 5 show stunting prevalence in indigenous and afrodescendant children from Latin American countries by wealth tertiles and place of residence, respectively. Brazil, Guyana, Paraguay and Suriname where excluded due to small sample sizes (fewer than 25 children in two or three tertiles of indigenous children. In Belize, Mexico, Nicaragua and Peru results are not shown for the richest tertile, also due to small sample size. In all countries and ethnic groups, there were monotonic decreases in stunting prevalence from the poorest to the wealthiest tertiles. Stunting prevalence among indigenous children, in all countries, was higher than reference children from the same wealth tertile (Fig. 4a). In several groups, “bottom inequality” patterns were observed, with children in the poorest tertile showing markedly higher prevalence than those in the other two tertiles; this was observed for indigenous children from Colombia and Honduras, and reference-group children from Honduras, Mexico and Peru. In contrast, afrodescendant children presented a lower prevalence of stunting than the reference groups in the same tertile, except for Brazil and Suriname. With few exceptions such as Colombia and Suriname, the widths of the inequality gaps in afrodescendant and reference children were quite similar (Fig. 4b). In all countries, the slope index for absolute inequality was significantly negative in all ethnic groups, except for reference children from Brazil and Suriname and afrodescendant children from Ecuador and Guyana.

Figure 5 shows that rural children tended to be more stunted than urban children. Rural and urban indigenous children were more stunted than reference children in the same categories (Fig. 5a). Additionally, the inequality gap among indigenous children was wider than among the reference children. Tests for interaction between residence and ethnicity (indigenous versus reference) were significant in all countries except in Brazil (*p* = 0.1).

In contrast, rural and urban afrodescendant children in each tertile were less stunted than the reference groups. Urban-rural gaps in stunting prevalence among afrodescendant children were wider in Colombia and Suriname, whereas for rural and urban reference children the larger gaps were observed in Belize, Ecuador and Honduras (Fig. 5b). Interactions between residence and ethnicity (afrodescendants versus reference group) were significant in all countries except for Brazil (*p* = 0.6) and Guyana (*p* = 0.1).

In order to investigate potential determinants of stunting in indigenous children, we examined indicators related to breastfeeding and complementary feeding at the age range of 6–23 months by country, when linear growth faltering is most intense (Table 2). Although indigenous children had consistently higher prevalence of breastfeeding in all countries, they also had lower frequencies of the three indicators of adequate complementary feeding than among reference children, except in Honduras. The very low proportions of children in either group with minimum acceptable diets in some countries are noteworthy. There were no consistent differences between afrodescendants and the reference group in terms of dietary patterns (Additional file 1: Supplementary Table 2).

**Table 2:** Breastfeeding and complementary feeding among children aged 6–23 months among indigenous and reference group

Country/year	Ethnic group	N	Breastfeeding	Minimum dietary diversity	Minimum meal frequency	Minimum acceptable diet
			% (95% CI)	% (95% CI)	% (95% CI)	% (95% CI)
Belize 2015	Indigenous	114	80.6 (72.0; 87.0)	56.5 (45.2; 67.1)	14.7 (9.1; 23.0)	1.6 (0.4; 6.8)
	Reference	348	58.0 (51.4; 64.4)	66.1 (59.8; 71.9)	28.2 (23.0; 34.1)	3.7 (1.9; 6.9)
Bolivia 2018	Indigenous	1428	78.0 (75.3; 80.5)	66.7 (63.3; 69.9)	–	–
	Reference	1030	65.9 (62.1; 69.4)	77.8 (74.3; 81.0)	–	–
Brazil 2006	Indigenous	31	70.6 (26.7; 94.1)	–	–	–
	Reference	444	40.6 (33.2; 48.4)	–	–	–
Colombia 2010	Indigenous	784	71.5 (65.3; 76.9)	59.4 (52.8; 65.7)	–	–
	Reference	3616	54.9 (52.8; 56.8)	74.5 (72.8; 76.2)	–	–
Ecuador 2012	Indigenous	494	80.2 (74.5; 84.9)	52.7 (45.7; 59.5)	8.5 (5.3; 13.6)	1.7 (0.9; 3.4)
	Reference	2491	58.7 (55.9; 61.6)	70.4 (67.0; 73.7)	28.9 (26.1; 31.8)	16.5 (13.9; 19.4)
Guatemala 2014	Indigenous	1524	88.1 (86.1; 89.9)	59.0 (55.8; 62.1)	82.1 (79.6; 84.3)	51.0 (47.8; 54.2)
	Reference	1975	70.5 (67.9; 73.0)	65.7 (62.9; 68.3)	82.8 (80.7; 84.7)	53.0 (50.3; 55.7)
Guyana 2014	Indigenous	225	78.7 (71.5; 84.4)	46.3 (38.6; 54.3)	–	–
	Reference	542	43.6 (37.6; 49.7)	55.9 (50.1; 61.6)	–	–
Honduras 2011	Indigenous	542	76.3 (71.7; 80.4)	67.4 (62.2; 72.1)	88.5 (85.1; 91.2)	56.8 (51.5; 61.8)
	Reference	2518	65.3 (63.1; 67.5)	67.6 (65.4; 69.8)	85.6 (83.8; 87.3)	54.2 (51.6; 56.8)
Mexico 2015	Indigenous	227	63.2 (47.2; 76.8)	55.6 (41.7; 68.6)	63.7 (51.2; 74.6)	21.3 (13.7; 31.7)
	Reference	2084	41.3 (37.2; 45.5)	73.7 (69.5; 77.5)	76.7 (73.3; 79.8)	18.8 (16.0; 22.0)
Nicaragua 2006	Indigenous	106	73.3 (59.7; 80.6)	–	–	–
	Reference	1730	62.2 (59.1; 65.2)	–	–	–
Paraguay 2016	Indigenous	102	84.2 (71.3; 92.0)	25.7 (17.7; 35.8)	31.6 (20.9; 44.8)	10.6 (5.2; 20.5)
	Reference	1341	45.2 (41.5; 49.0)	71.9 (68.5; 75.1)	71.8 (68.4; 74.9)	16.6 (14.1; 19.4)
Peru 2016	Indigenous	561	89.1 (85.7; 91.7)	69.9 (65.0; 74.5)	–	–
	Reference	5634	74.3 (72.8; 75.8)	83.7 (82.3; 84.9)	–	–
Suriname 2010	Indigenous	63	53.7 (37.6; 69.0)	–	25.7 (15.0; 40.3)	–
	Reference	335	23.8 (19.3; 28.9)	–	63.3 (57.3; 69.0)	–

## Discussion

In Latin America, stunting prevalence fell from 23.7% in 1990 to 13.5% in 2010, a 43% reduction, and prevalence is projected to fall to 10% by 2020 [42]. However, overall progress may hide important within-country inequalities. In the 12 countries included in the present analyses, the median prevalence among indigenous children was 31.9%, being as high as 61.4% in Guatemala.

Several studies from the Latin American region used data from national surveys to compare stunting prevalence among indigenous and non-indigenous children at a given point in time. An analysis of data from the 1990’s in Bolivia, Colombia, Ecuador and Peru had already signaled the higher prevalence of stunting among indigenous than among non-indigenous children, the latter group including afrodescendants [15]. A PAHO report using data from 2002 to 2008 showed similar results from Bolivia, Ecuador, Guatemala, and Peru [16]. The most recent overview provided data collected since 2018 on seven countries (Bolivia, Ecuador, Guatemala, México, Nicaragua, Panamá and Paraguay), confirming the higher prevalence of stunting among indigenous children [13]. Similar patterns were described in single-country reports on Brazil [14] and Guatemala [43].

Other authors described time trends in stunting in the indigenous children for Guatemala (1998–2008) [17], Mexico (1988–2012) [18] Bolivia (2003–2008) [11], Brazil (1996–2006) [11] and Peru (2007–2012) [11]. In all of these studies, stunting prevalence fell for both indigenous and non-indigenous children, yet the former continued to present significantly higher prevalence than the latter. The exception was Brazil where the samples for indigenous children were small in 1996 and 2006, and the differences were not significant [11]. A separate national survey restricted to indigenous children in 2008–2009 showed substantially a higher prevalence of stunting than was observed for non-indigenous children in the 2006 national survey [14].

None of the above studies reported on analyses that were stratified by ethnicity and either wealth or residence, nor that separated afrodescendants from the reference group, nor that attempted to explain the ethnic gap by adjusting for wealth or residence.

Our multicountry analyses confirmed that, in all countries studied, indigenous children tended to be more stunted than reference children in the crude analyses. In Brazil and Suriname, the differences in stunting prevalence were not significant, but the differences in terms of mean length/height-for-age curves reached statistical significance for all countries suggesting that linear growth faltering among indigenous children is a population-level problem. We also showed that indigenous children were also poorer and more likely to live in rural areas.

Our findings need to be interpreted in light of a conceptual model for explaining the etiology of undernutrition [1]. In such a model, ethnicity represents a distal or structural determinant of health [44], as it influences intermediate determinants such as income, wealth, education and place of residence, which is confirmed by our results showing that indigenous children tended to be poorer and more rural than other children. At the proximal level of determination, linear growth faltering is influenced by diets, childcare and the incidence of illness, particularly infections [1, 45].

The unadjusted analyses show the full effect of ethnicity, whereas adjusted models show how much of an effect remains after considering wealth and place of residence, which may be regarded as intermediate determinants, driven by ethnicity and, in turn, affecting nutritional status [46]. Even after adjustment for wealth and place of residence, stunting prevalence remained higher among indigenous children in eight of the 13 countries, suggesting that the differences cannot be explained by these two covariates. Moreover, the well-known association between wealth and stunting was observed within each ethnic group [5].

These findings on stunting are consistent with a biocultural framework which explains how *“the devastating effects of colonization, the loss of ancestral land, and language and cultural barriers for access to health care are among the most salient themes characterizing the poor health situation of indigenous people”* [47]. There is ample literature on the high rates of several infectious disease among indigenous people [12, 47, 48]. Survey data from several Latin American countries showed lower health care coverage among indigenous women and children than for the rest of the population, even after adjusting for wealth and residence [34]. Lower coverage has been associated with an organization of health services that is insensitive to the need for intercultural health care [49].

In addition to illness, diet is a key determinant of growth. Our analyses of dietary indicators of children aged 6–23 months showed that although indigenous children were more likely to be breastfed, they tended to have worse complementary diets than the reference group, particularly in terms of dietary diversity. Specifically, indigenous children receive breastmilk with low-quality complementary foods in an age range when breastfeeding alone is insufficient to meet the nutritional needs of the infant for optimal growth. This finding is in agreement with studies on food security in the Latin American region [13]. Our analyses of complementary diets relied upon the 2008 definitions adopted by WHO [50], which are currently being updated and revised [51]. For example, the updated version of the minimum dietary diversity indicator will require the consumption of five from eight different food groups, as opposed to the present requirement of four out of seven groups. This will lead to lower proportions of children being classified as adequately fed, but differences between ethnic groups will likely remain. Nevertheless, the same criteria were used for all children so that the comparisons could be valid.

Our results reflect the social exclusion pattern that systematically affects indigenous people in Latin American, and that persists in spite of progress in indicators reflecting health and socioeconomic conditions [10]. Discrimination against indigenous populations, affecting their access to health and other services, and their lack of political representation also play a role in explaining the higher prevalence of stunting in this population [52]. Language and culture are important issues as well, being essential components of the identity and worldviews of indigenous peoples that exert a major influence on their health and nutrition [53]. Specifically, language often represents a significant barrier to the communication between indigenous patients and health-care providers, thus restricting access to careseeking and limiting the effectiveness of patient-provider interactions [54]. In short, social exclusion, discrimination, language and culture are key drivers of ethnic inequalities in nutrition and health indicators [10, 11, 54]. Therefore, a comprehensive and inclusive intercultural health model should be a key component of health services in the Latin American countries [49].

Our comparisons of linear growth in afrodescendants and the reference group showed few differences between the two groups. In five out of the seven countries with data, afrodescendants were more likely to be urban, and in half of these countries they were richer than the reference group. After adjustment for wealth and residence, two countries – Colombia and Ecuador – showed a lower prevalence of stunting in afrodescendants than in the reference children, and no country showed higher prevalence among the latter. The published literature on this comparison is scarce; we were able to find a study from Colombia reporting that adult afrodescendants were taller than individuals classified as indigenous or “others” [55], and a birth cohort from Southern Brazil where one-year old children born to white and black mothers had similar prevalence of stunting [56].

Our analyses have a number of limitations. Although standardized child health and nutrition surveys are available for most countries in the Latin American region, several surveys failed to collect information on either anthropometry or ethnicity (i.e., Costa Rica, Dominican Republic and Panama). Moreover, sample sizes for indigenous children were very small, as was the case in Brazil and Paraguay, and some surveys were carried out before 2010, such as in Bolivia, Brazil and Nicaragua.

An important limitation is the nature of the information on ethnicity, which is recognizably an important issue in all studies of ethnicity and health based on self-classification [12]. In Paraguay and Peru, the language spoken at home was used as a proxy variable, but it is possible that indigenous families also speak Spanish. In Paraguay, Guarani is taught in all public schools in the country, so many non-indigenous women speak both Spanish and Guarani. In Brazil the information was on self-reported skin color, with a single specific category for “indigenous. This represents the standard classification endorsed by the Brazilian Institute of Geography and Statistics (https://ibge.gov.br). In addition, due to sample size considerations, a single category was employed for indigenous children, even though in several countries this group includes a broad variety of indigenous communities, each with unique world views, languages, traditions, feeding practices and traditional healing systems. This also applies to afrodescendants to some extent, as there were countries that could have grouped very different cultures into one category [19]. It should be noted that survey sampling frames were not designed specifically with ethnicity in mind. Nevertheless, this does not preclude the comparisons among ethnic groups, just as it is possible to stratify results by wealth or education even though these dimensions did not represent specific sampling domains in surveys.

Due the complexity of ethnic group classifications [12], the proportions of the survey samples classified in each group might differ from those measured in population censuses or in other surveys. In addition, differential fertility may result in larger proportions of children being classified as indigenous or afrodescendants than would be the case for national censuses that count all individuals. Different types of questions and categories used in each type of survey may lead to the aforementioned discrepancies in the survey samples and the small number of observations when the information is disaggregated into subgroups [34]. Thus, our results must be interpreted carefully, although the fact that our classification showed large and mostly consistent results for indigenous children suggests that the categories included in the analyses are able to discriminate population subgroups.

The quality of anthropometric measurements in routine surveys has been questioned [57]. In Additional file 1: Supplementary Table 3, we show that this is unlikely to have biased our results, because nearly all standard deviations of height for age (expressed in Z scores) ranged from 1.0 to 1.5, which is the expected range in high-quality studies [35].

Lastly, given intense miscegenation, which is characteristic in the Latin American region, the reference group includes substantial proportions of children with mixed ethnic backgrounds, whose families did not identify themselves as being indigenous or afrodescendants. In spite of the above limitations, the present analyses comprise the most comprehensive report on ethnic differences in linear growth failure and feeding practices across several countries in the Latin American region, relying upon standardized survey methods and analytical approaches.

## Conclusion

We consistently found a higher prevalence of stunting in indigenous children, whereas afrodescendant and reference children had similar levels in most countries. Our findings persisted after adjustment for wealth and residence, suggesting that other factors such as a lack of access to resources, likely related to discrimination or cultural inadequacy, play a role in the determination of growth faltering among indigenous children. In terms of dietary patterns, indigenous children aged 6–23 months were more likely to be breastfed, but with poor complementary feeding. However, no consistent differences were found between afrodescendants and the reference group. Continued monitoring of ethnic inequalities, as required by SDG 17.18, is essential to assess the impact of inclusive policies in the Latin American region and to guide future policy and programmatic initiatives.

## Supplementary information

**Additional file 1: Table S1.** Data sources and definitions of ethnicity for the countries included in the analyses. **Table S2.** Breastfeeding and complementary feeding among children aged 6–23 months among afrodescendant and reference group. **Table S3.** Mean and standard deviation of height for age z-score by country and ethnic group.

**Additional file 2: Figure S1.** Distribution of indigenous, afrodescendants and reference children according to national wealth tertiles. **Figure S2.** Distribution of indigenous, afrodescendants and reference children according to place of residence

## Data Availability

Not applicable.
