# Supplementary material for: Ethnic inequalities in child stunting and feeding practices: results from surveys in thirteen countries from Latin America
Source: Int J Equity Health. 2020 Apr 9;19:53. doi: 10.1186/s12939-020-01165-9 (PMC7147069; doi:10.1186/s12939-020-01165-9)
Supplement: Supplementary file 1 — Additional file 1: Table S1. Data sources and definitions of ethnicity for the countries included in the analyses. Table S2. Breastfeeding and complementary feeding among children aged 6–23 months among afrodescendant and reference group. Table S3. Mean and standard deviation of height for age z-score by country and ethnic group. [file 12939_2020_1165_MOESM1_ESM.docx]

**Supplementary table 1.** Data sources and definitions of ethnicity for the countries included in the analyses.

| **Country/year** | **Information** | **Ethnic group** | **Definition (as in original questionnaire)** |
| --- | --- | --- | --- |
| Belize  2015  MICS | Ethnicity  (head of household) | Reference | Mestizo, others |
|  |  | Indigenous | Maya |
|  |  | Afrodescendant | Creole, Garifuna |
| Bolivia  2008  DHS | Ethnicity  (woman) | Reference | No ethnic affiliation declared |
|  |  | Indigenous | Quechua, Aymara, Guarani, other indigenous group |
|  |  | Afrodescendant | Not available |
| Brazil  2006  PNDS | Ethnicity/skin color  (woman) | Reference | White |
|  |  | Indigenous | Indigenous |
|  |  | Afrodescendant | Brown or black |
| Colombia  2010  DHS | Ethnicity  (woman) | Reference | Other |
|  |  | Indigenous | Native colombian |
|  |  | Afrodescendant | Raizal from archipelago, Palanquero from San Basilio, black/mulato/Afro-Colombian/Afrodescendant |
| Ecuador  2012  ENSANUT | Ethnicity  (woman) | Reference | White, mestizo |
|  |  | Indigenous | Indigenous |
|  |  | Afrodescendant | Black/Afro-equatorian/Mulato |
| Guatemala  2014  DHS | Ethnicity  (woman) | Reference | Ladino/mestizo |
|  |  | Indigenous | Maya, Xinca |
|  |  | Afrodescendant | Not available |
| Guyana  2014  MICS | Ethnicity  (head of household) | Reference | Mixed race/East Indian |
|  |  | Indigenous | Amerindian |
|  |  | Afrodescendant | African |
| Honduras  2011  DHS | Ethnicity  (woman) | Reference | No ethnic affiliation declared |
|  |  | Indigenous | Tolupan, Pech (Paya), Misquito, Nahoa, Lenca, Tawaka (Sumo), Maya chorti |
|  |  | Afrodescendant | Garifuna, black English |
| Mexico  2015  MICS | Ethnicity  (head of household) | Reference | Non-indigenous household |
|  |  | Indigenous | Indigenous household |
|  |  | Afrodescendant | Not available |
| Nicaragua  2006  RHS | Ethnicity  (woman) | Reference | Mestizo from the Caribbean coast, no ethnicity  declared |
|  |  | Indigenous | Rama, Mayangna-Sumu, Miskitu, Ulwa, Xiu-Sutiava,  Nahoa-Nicarao, Chorotega-Nahua-Mange,  Cacaopera-Matagalpa |
|  |  | Afrodescendant | Not available |
| Paraguay  2016  MICS | Language  (head of household) | Reference | Spanish or Spanish/Guarani* |
|  |  | Indigenous | Guarani-only |
|  |  | Afrodescendant | Not available |
| Peru  2016  DHS | Language  (household) | Reference | Spanish |
|  |  | Indigenous | Quechua, Aymara, other indigenous |
|  |  | Afrodescendant | Not available |
| Suriname  2010  MICS | Ethnicity  (head of household) | Reference | Creole/Indian/Javanese /mixed race |
|  |  | Indigenous | Indigenous/ Amerindian |
|  |  | Afrodescendant | Afrodescendant or black |

(*) Because Guarani is taught in all public schools in the country, many non-indigenous women speak both Spanish and Guarani

**Supplementary table 2.** Breastfeeding and complementary feeding among children aged 6-23 months among afrodescendant and reference group.

| **Country/year** | **Ethnic group** | **N** | **Breastfeeding** | **Minimum dietary diversity** | **Minimum meal frequency** | **Minimum acceptable diet** |
| --- | --- | --- | --- | --- | --- | --- |
|  |  |  | **% (95% CI)** | **% (95% CI)** | **% (95% CI)** | **% (95% CI)** |
| Belize  2015 | Afrodescendant | 200 | 48.9 (40.3; 57.6) | 69.8 (60.9; 77.4) | 32.3 (24.6; 41.2) | 2.1 (0.8; 5.5) |
|  | Reference | 348 | 58.0 (51.4; 64.4) | 66.1 (59.8; 71.9) | 28.2 (23.0; 34.1) | 3.7 (1.9; 6.9) |
| Brazil 2006 | Afrodescendant | 830 | 50.9 (44.0; 57.8) | - | - | - |
|  | Reference | 444 | 40.6 (33.2; 48.4) | - | - | - |
| Colombia 2010 | Afrodescendant | 621 | 50.2 (45.5; 55.0) | 63.5 (58.9; 67.8) | - | - |
|  | Reference | 3616 | 54.9 (52.8; 56.8) | 74.5 (72.8; 76.2) | - | - |
| Ecuador 2012 | Afrodescendant | 128 | 58.1 (44.0; 70.9) | 69.7 (57.4; 79.8) | 23.2 (14.4; 35.3) | 5.9 (3.3; 10.1) |
|  | Reference | 2491 | 58.7 (55.9; 61.6) | 70.4 (67.0; 73.7) | 28.9 (26.1; 31.8) | 16.5 (13.9; 19.4) |
| Guyana 2014 | Afrodescendant | 263 | 54.9 (46.9; 62.7) | 49.8 (42.4; 57.3) | - | - |
|  | Reference | 542 | 43.6 (37.6; 49.7) | 55.9 (50.1; 61.6) | - | - |
| Honduras 2011 | Afrodescendant | 87 | 50.0 (36.4; 63.5) | 75.5 (63.4; 84.6) | 83.1 (68.1; 91.9) | 50.4 (37.2; 63.5) |
|  | Reference | 2518 | 65.3 (63.1; 67.5) | 67.6 (65.4; 69.8) | 85.6 (83.8; 87.3) | 54.2 (51.6; 56.8) |
| Suriname 2010 | Afrodescendant | 652 | 28.6 (24.5; 33.0) | - | 53.9 (49.0; 58.8) | - |
|  | Reference | 335 | 23.8 (19.3; 28.9) | - | 63.3 (57.3; 69.0) | - |

**Supplementary table 3.** Mean and standard deviation of height for age z-score by country and ethnic group

| **Country/Year** | **Height for age Z-score** | | |
| --- | --- | --- | --- |
|  | **Indigenous** | **Afrodescendants** | **Reference** |
| Belize 2015 | -1.50 ± 1.2 | -0.44 ± 1.2 | -0.92 ± 1.1 |
| Bolivia 2008 | -1.45 ± 1.3 | NA | -0.87 ± 1.3 |
| Brazil 2006 | -0.72 ± 1.6 | -0.47 ± 1.5 | -0.21 ± 2.0 |
| Colombia 2010 | -1.48 ± 1.1 | -0.63 ± 1.2 | -0.75 ± 1.1 |
| Ecuador 2012 | -1.67 ± 1.5 | -0.75 ± 1.3 | -1.12 ± 1.3 |
| Guatemala 2014 | -2.31 ± 1.1 | NA | -1.56 ± 1.2 |
| Guyana 2014 | -1.15 ± 1.5 | -0.09 ± 1.4 | -0.30 ± 1.4 |
| Honduras 2011 | -1.52 ± 1.3 | -0.76 ± 1.2 | -1.19 ± 1.2 |
| Mexico 2015 | -1.41 ± 1.2 | NA | -0.74 ± 1.1 |
| Nicaragua 2006 | -1.37 ± 1.4 | NA | -1.09 ± 1.3 |
| Paraguay 2016 | -1.37 ± 1.2 | NA | -0.27 ± 1.1 |
| Peru 2016 | -1.65 ± 1.0 | NA | -0.85 ± 1.0 |
| Suriname | -0.89 ± 1.2 | -0.59 ± 1.3 | -0.40 ± 1.3 |
