# Supplementary material for: Ethnic inequalities in child stunting and feeding practices: results from surveys in thirteen countries from Latin America
Source: Int J Equity Health. 2020 Apr 9;19:53. doi: 10.1186/s12939-020-01165-9 (PMC7147069; doi:10.1186/s12939-020-01165-9)
Supplement: Supplementary file 2 — Additional file 2: Figure S1. Distribution of indigenous, afrodescendants and reference children according to national wealth tertiles. Figure S2. Distribution of indigenous, afrodescendants and reference children according to place of residence [file 12939_2020_1165_MOESM2_ESM.docx]

**Supplementary figure 1.** Distribution of indigenous, afrodescendants and reference children according to national wealth tertiles **
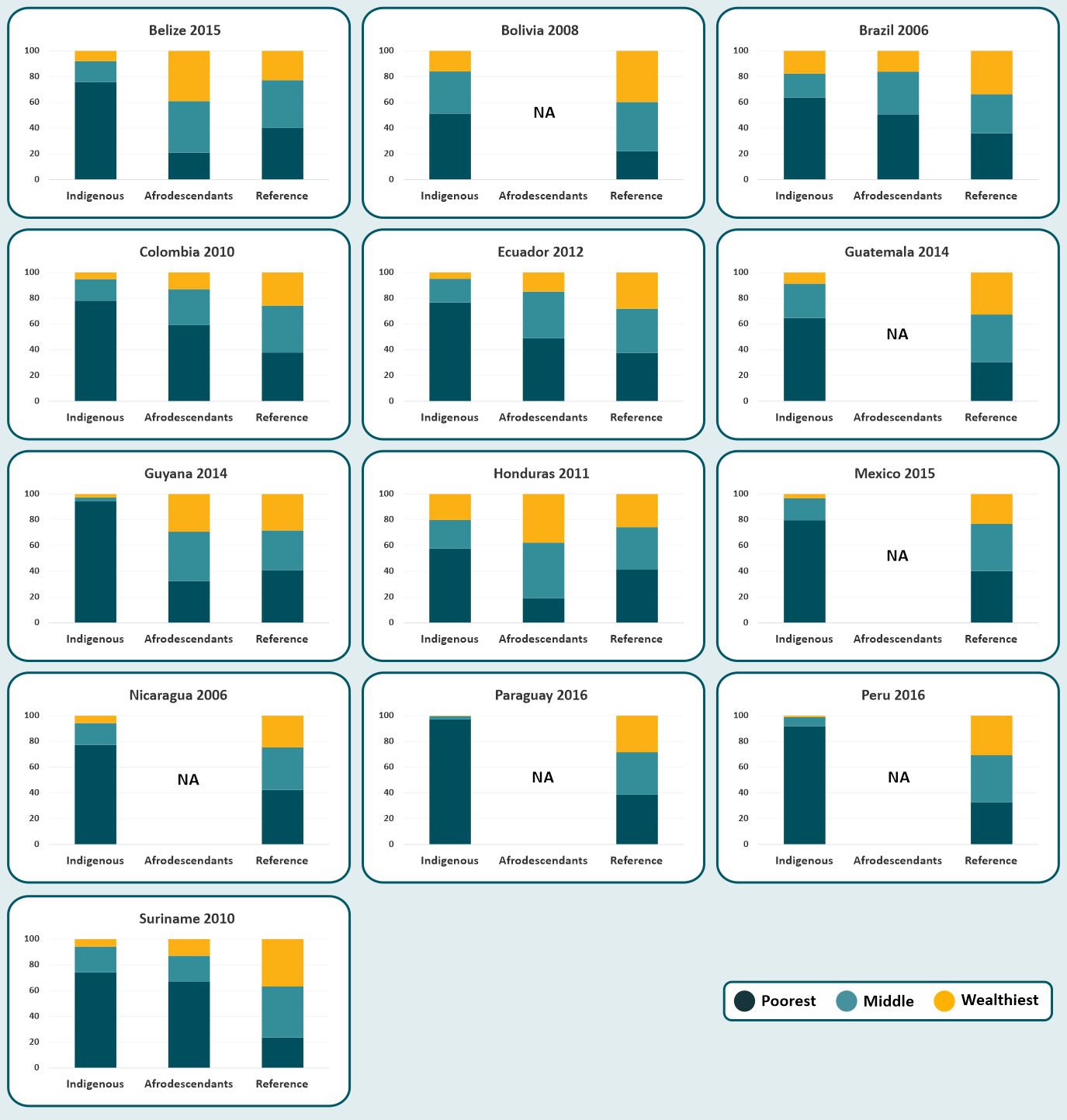
**

**
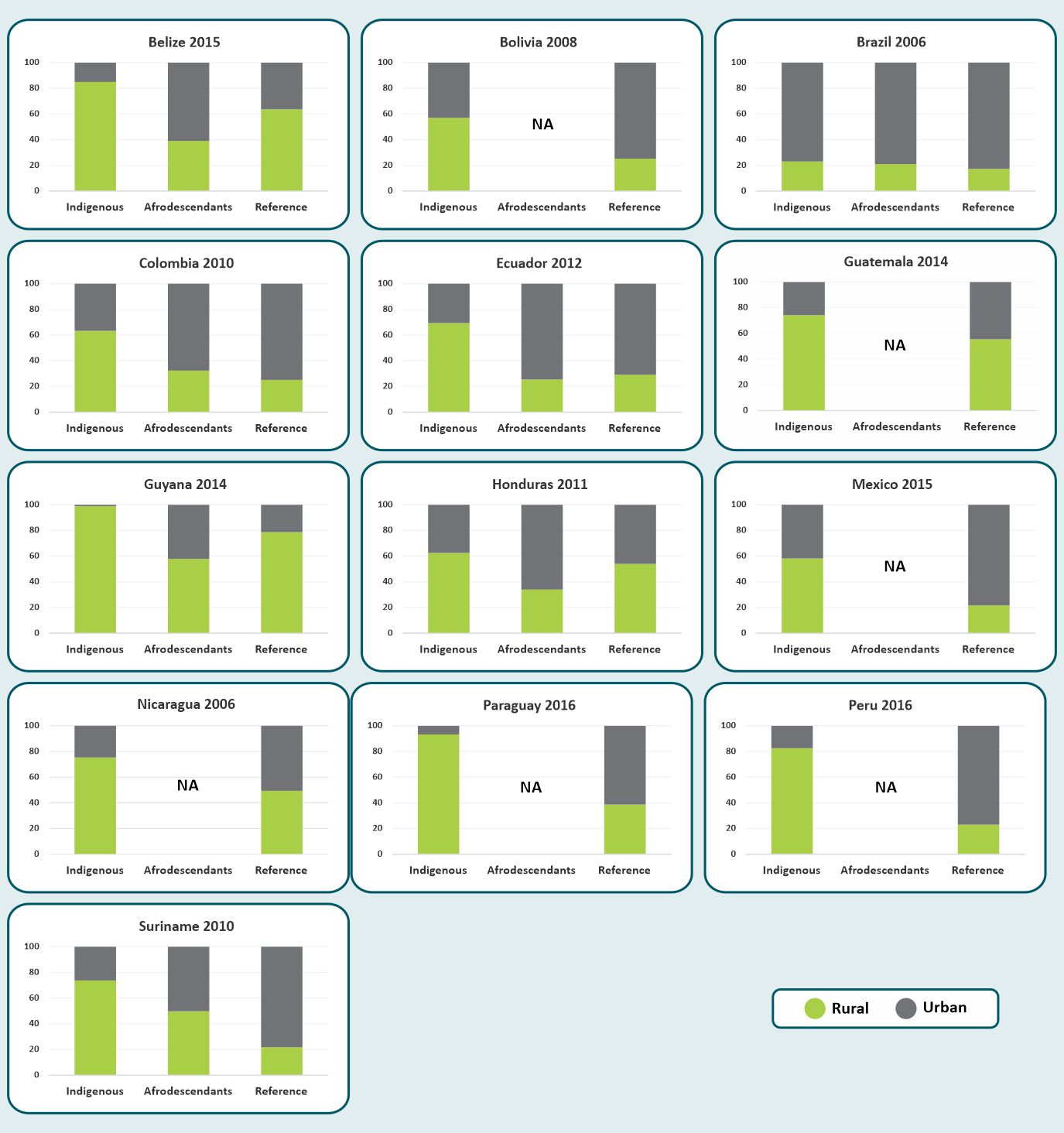
Supplementary figure 2.** Distribution of indigenous, afrodescendants and reference children according to place of residence
